# Infected urachal remnants: an unusual presentation

**DOI:** 10.1259/bjrcr.20150226

**Published:** 2016-05-14

**Authors:** Victor Hugo Ramos Pacheco, Yolanda Saldaña Dominguez, Alicia Maria del Consuelo Cervantes Sánchez

**Affiliations:** ^1^ Department of Radiology and Image, Hospital General De México, “Dr. Eduardo Liceaga”, Mexico City, Mexico; ^2^ Private practice in Mexico City, Mexico City, Mexico

## Abstract

The urachus is the remnant of the cloaca and allantois, and attaches the umbilicus to the bladder dome. Urachal anomalies are symptomatically identified during childhood. It rarely occurs in adults, making diagnosis difficult. We present and discuss the case of an infected patent urachus in a 30-year-old male.

## Summary

The urachus is a midline tubular structure that extends upward from the anterior dome of the bladder towards the umbilicus. The urachus involutes before birth, remaining as a fibrous band with no known function.^1^ Remnants of the tract may present as patent urachus, vesicourachal diverticulum, urachal sinus or urachal cyst.^2^ Because urachal remnant diseases are uncommon, the signs and symptoms are non-specific and the diagnosis is not easily made. Ultrasound and CT scan play an important role in the diagnosis of this pathology.

## Case report

A 30-year-old male presented with history of lower abdominal pain and periumbilical erythema, which had been persisting for 15 days, with nausea, vomiting, fever and umbilical discharge for the past 8 days. Physical examination revealed a temperature of 38.7°C. His abdomen was soft and there was umbilical discharge with erythema and a tender umbilical mass. Laboratory tests revealed leucocytosis of 22,000 mm^–3^. A urinalysis was within normal ranges.

**Figure 1.**
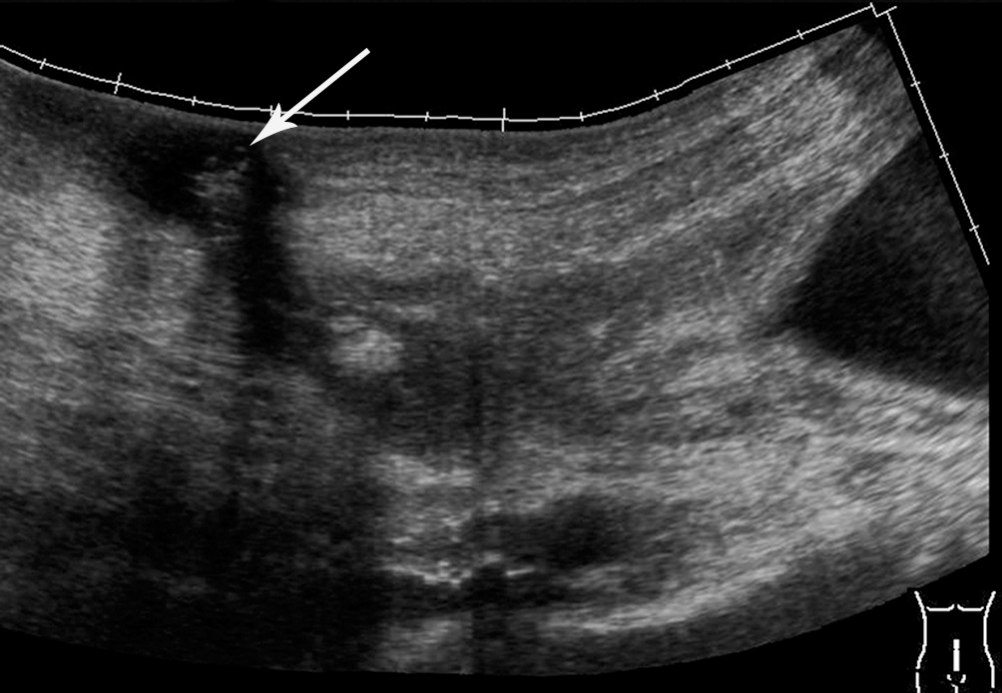
Panoramic image of the abdominal ultrasound showing patent urachus, with presence of gas (arrow).

**Figure 2.**
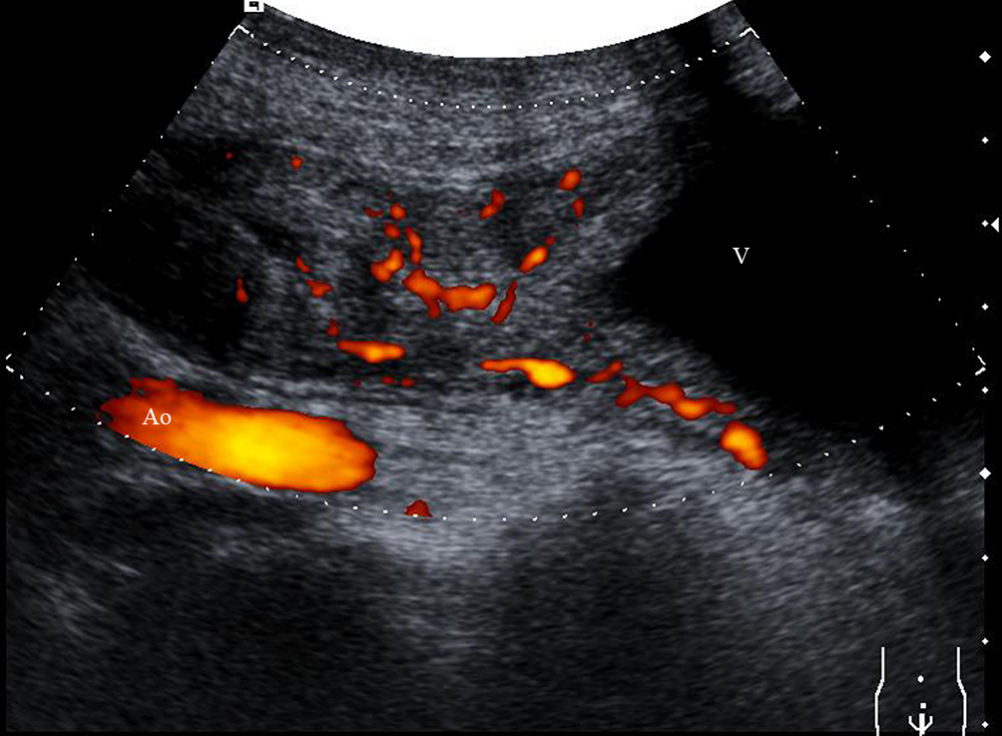
Longitudinal scan colour power Doppler showing hypoechoic lesion anterior to the aorta (Ao) with increased vascularity; the triangular aspect of the vesical dome (V) is noteworthy.

**Figure 3.**
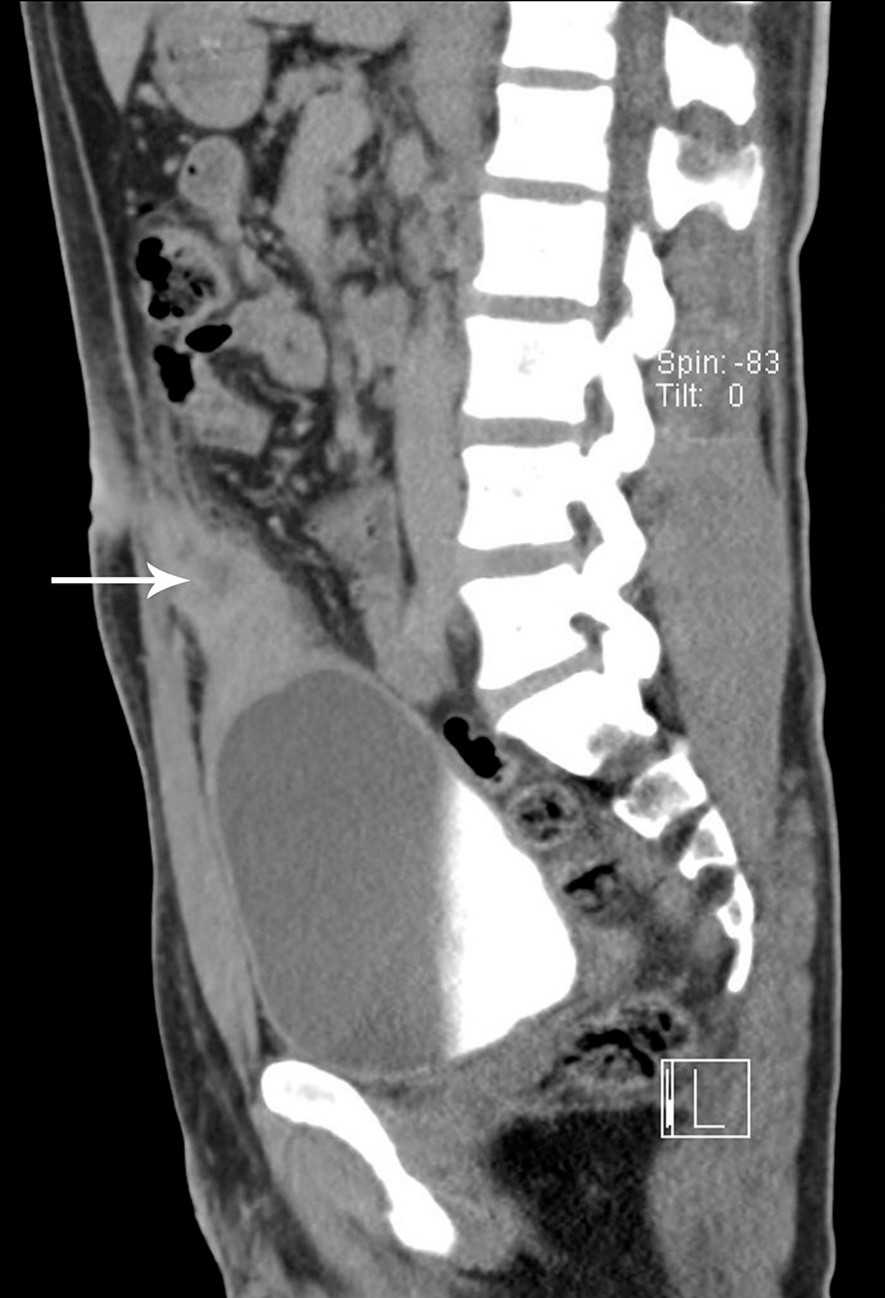
Sagittal CT image showing hypodense mass, anterior and superior to the vesical dome extending to the umbilicus. The presence of liquid collection adjacent to the umbilicus (arrow) is significant.

**Figure 4.**
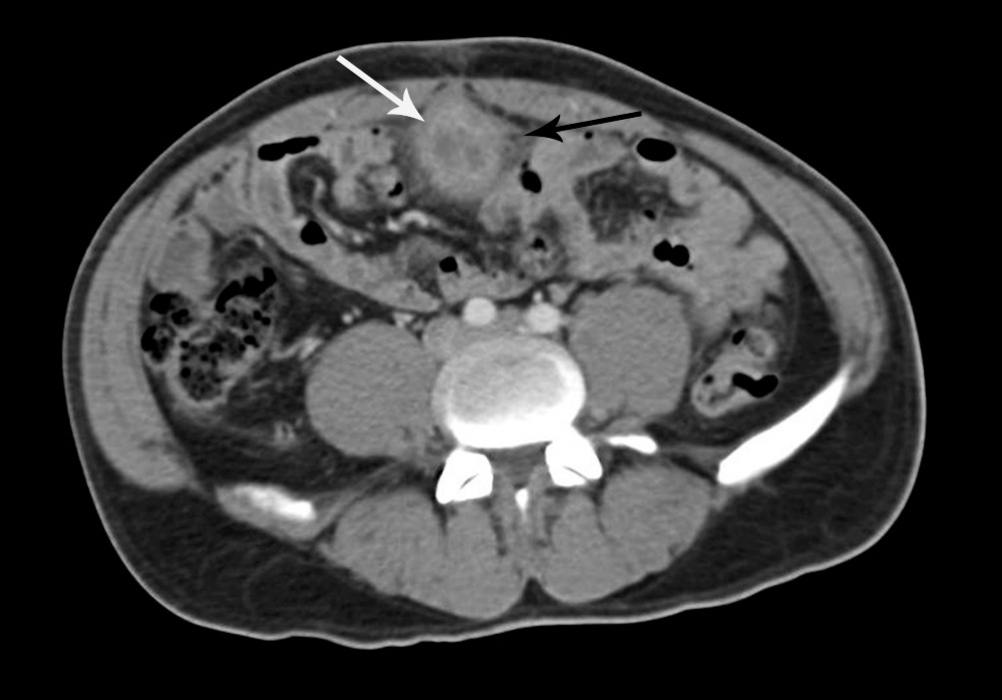
Axial CT image showing abscess formation in patent urachus posterior to the abdominal muscles. The rim enhancement with contrast (white arrow) and the fat stranding (black arrow) are significant.

Ultrasonography revealed the presence of a heterogeneous mass of size 7.2 × 3.2 cm in the midline extending from the anterior and superior wall of the urinary bladder to the umbilicus with presence of gas (Figure 1). Colour power Doppler showed increased vascularity (Figure 2). CT scan of the abdomen confirmed the presence of a tubular mass extending from the umbilicus to the dome of the urinary bladder (Figure 3). Contrast CT scan showed rim enhancment, with low density of the fluid collection and fat stranding (Figure 4). The cystoscopy showed an inflamed area of the bladder dome with poor purulent drainage. The patient received antibiotic therapy for a week and the mass was removed by surgical excision. Histopathological evaluation revealed an umbilical–urachal sinus with xanthogranulomatous inflammation. The patient presented satisfactory resolution of his symptoms.

## Discussion

The urachus is a vestigial structure that develops from two embryological structures: the cloaca (an extension of the urogenital sinus) and the allantois (derived from yolk sac). During the fourth and fifth months of gestation, the foetal bladder descends into the pelvis and the allantois lumen is obliterated to form the urachus.^1^ This structure closes by the third trimester of gestation or after birth, forming the median umbilical ligament.^3^ The urachus lies in the space of Retzius, between transversalis fascia anteriorly and the peritoneum posteriorly. Histologically, it is composed of all the three bladder layers, the innermost layer being lined with transitional epithelium in 70% and columnar epithelium in 30% of cases.^4^

**Figure 5.**
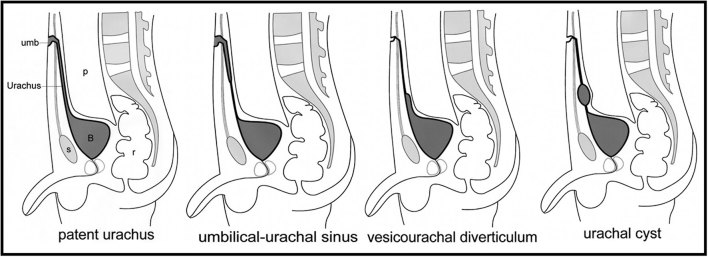
Diagram showing the four types of urachal anomalies. Patent urachus or urachal fistula, umbilical–urachal sinus, vesicourachal diverticulum and urachal cyst. B, bladder; p, peritoneal cavity; r, rectum; s, symphysis pubis; umb, umbilicus. Adapted from Yu et al^1^ with permission from The Radiological Society of North America.

The incomplete involution of urachus results in four types of abnormalities: patent urachus, umbilical–urachal sinus, vesicourachal diverticulum and urachal cyst (Figure 5).^1^ These anomalies are twice as common in males, and the most common presentation is the patent urachus in about 50% of cases.^5^ The patent urachus usually presents during the neonatal period with urinary discharge from the umbilicus, and are extremely rare in adults. An umbilical–urachal sinus, vesicourachal diverticulum or urachal cyst is generally asymptomatic and can reopen in association with pathological conditions.^2^

Luchtman et al^6^ showed that children are more likely to have an infected urachal cyst, while adults are more likely to have an infected urachal sinus.^6^ Infection may spread through the blood, lymphatic or directly from the bladder. The cultured organisms include *Escherichia coli, Enterococcus faecium, Klebsiella pneumoniae, Proteus, Streptococcus viridians* and *Fusobacterium.* ^7^

The clinical manifestations of patent urachal pathologies are umbilical discharge, erythema and mass within the umbilicus. The abdominal pain can mimic other conditions such as appendicitis or Meckel’s diverticulum. The differential diagnoses include abscess, umbilical hernia, urachal carcinoma and other tumours.^3^

Urachal anomalies can be accurately demonstrated by both a CT scan and an ultrasound; other diagnostic studies include i.v. pyelography, cystography, cystoscopy and MR. Ultrasound is usually enough to diagnose an infected urachal remnant. Cacciarelli et al^8^ described the urachus as an elliptical, hypoechoic structure, with variable size in the middle of the anterosuperior surface of the urinary bladder.^8^ A CT scan can accurately confirm the type of urachal anomaly. However, differentiating between an infection and carcinoma of the urachus can be difficult; calcification occurs in 50–70% of urachal carcinomas and this data can help in the differential diagnosis.^1^

The treatment of an infected urachus should include antibiotics combined with drainage of the abscess, followed by total excision of the remnant. This treatment seems to be the most effective option for avoiding the future development of malignant disease.

## Learning points

1. The presence of mass in the midline, umbilical discharge and abdominal pain are useful in clinical suspicion of urachal anomalies.
2. The imaging studies confirm the diagnosis, help define the anatomical relationships of the lesions and are useful in differentiating between benign and malignant disease.
3. An understanding of the embryological failure, associated imaging findings and complications are important for early diagnosis.

## Consent

Informed consent has been provided by the patient.
